# Molecular Imaging of Aortic Aneurysm and Its Translational Power for Clinical Risk Assessment

**DOI:** 10.3389/fmed.2022.814123

**Published:** 2022-04-15

**Authors:** Vinamr Rastogi, Sanne J. M. Stefens, Judith Houwaart, Hence J. M. Verhagen, Jorg L. de Bruin, Ingrid van der Pluijm, Jeroen Essers

**Affiliations:** ^1^Department of Vascular Surgery, Erasmus University Medical Center, Rotterdam, Netherlands; ^2^Department of Molecular Genetics, Erasmus University Medical Center, Rotterdam, Netherlands; ^3^Department of Radiation Oncology, Erasmus University Medical Center, Rotterdam, Netherlands

**Keywords:** aortic aneurysm, aortic rupture, molecular imaging, biomarkers, disease models, animal, translational medical research

## Abstract

Aortic aneurysms (AAs) are dilations of the aorta, that are often fatal upon rupture. Diagnostic radiological techniques such as ultrasound (US), magnetic resonance imaging (MRI), and computed tomography (CT) are currently used in clinical practice for early diagnosis as well as clinical follow-up for preemptive surgery of AA and prevention of rupture. However, the contemporary imaging-based risk prediction of aneurysm enlargement or life-threatening aneurysm-rupture remains limited as these are restricted to visual parameters which fail to provide a personalized risk assessment. Therefore, new insights into early diagnostic approaches to detect AA and therefore to prevent aneurysm-rupture are crucial. Multiple new techniques are developed to obtain a more accurate understanding of the biological processes and pathological alterations at a (micro)structural and molecular level of aortic degeneration. Advanced anatomical imaging combined with molecular imaging, such as molecular MRI, or positron emission tomography (PET)/CT provides novel diagnostic approaches for *in vivo* visualization of targeted biomarkers. This will aid in the understanding of aortic aneurysm disease pathogenesis and insight into the pathways involved, and will thus facilitate early diagnostic analysis of aneurysmal disease. In this study, we reviewed these molecular imaging modalities and their association with aneurysm growth and/or rupture risk and their limitations. Furthermore, we outline recent pre-clinical and clinical developments in molecular imaging of AA and provide future perspectives based on the advancements made within the field. Within the vastness of pre-clinical markers that have been studied in mice, molecular imaging targets such as elastin/collagen, albumin, matrix metalloproteinases and immune cells demonstrate promising results regarding rupture risk assessment within the pre-clinical setting. Subsequently, these markers hold potential as a future diagnosticum of clinical AA assessment. However currently, clinical translation of molecular imaging is still at the onset. Future human trials are required to assess the effectivity of potentially viable molecular markers with various imaging modalities for clinical rupture risk assessment.

## Introduction

Aortic Aneurysms (AA) are pathological dilations of the aorta, in which the diameter is enlarged to over 50% of its original size or over 30 mm (1, 2). Depending on their location, ‘AAs’ can be classified into either thoracic (TAA), thoracoabdominal (ThAAA), and abdominal aortic aneurysms (AAA). ‘TAAs’ are located within the chest cavity and are subclassified into aneurysms involving the aortic root, ascending aorta, aortic arch and/or the descending thoracic aorta. ‘AAAs’ are naturally located below the diaphragm within the abdomen, usually presenting at an infrarenal level (3). As such, ThAAA are extensive aortic dilatations covering the aortic portion within both the chest and abdomen.

Whilst TAA has an estimated prevalence of 5.3 per 100,000 (0.0053%) within the Western world (4), AAA is more prevalent, affecting an estimate of 1.3–8.9% of males and 1.0–2.2% of females (5–8). Interestingly, for the development of TAAs, aortic dilatation of the root and ascending aorta are often attributed to single genetic defects, such as in Marfan syndrome (9) and Loeys-Dietz syndrome (10). This is in contrast to AAAs, in which lifestyle seems to have a major contribution to disease progression (11). Predominantly men above the age of 65 are at risk of developing AAA (11), and additional risk factors besides a genetic predisposition include those similar to other cardiovascular diseases such as smoking (12), age, hypertension and obesity (13).

Aortic aneurysms are fatal upon rupture and therefore life-threatening, elucidating the necessity of timely intervention. This has resulted in screening of moderate risk populations (males aged above 65 years with cardiovascular risk profile) in countries including England and Sweden, leading to a reduction in all-cause mortality (14), whilst cost-effectiveness is questioned (15). Nevertheless, AA diagnosis in most countries remains a concomitant incidental finding, as only a small fraction of AAs leads to symptoms. After detection of aortic aneurysmal disease, clinical risk assessment of rupture is only done by measuring maximum diameter resulting in a one-size-fits-all threshold for repair set at 5.0–5.5 cm in various clinical guidelines, as no better method is currently available (1, 2, 16, 17). However, a recent study by Tan et al. reported that though these clinical practice guidelines were considered of adequate methodological quality to recommend their use in clinical practice, these still showed areas for improvement, potentially through performing economic analysis and trial application of recommendations (18). Furthermore, although aortic diameter is correlated with rupture risk, the expansion rate of AA differs greatly per individual (19), is discontinuous (20), and therefore is no exclusive parameter to deliver a highly accurate risk of rupture. Furthermore, studies on ruptured AAAs demonstrated that large variations in diameters were found with a considerable number of patients rupturing their AAAs at diameters >7–8 cm (21, 22). Therefore, a more patient tailored approach seems to be desirable.

Currently, AA patients are increasingly treated with minimally invasive, endovascular aneurysm repair (EVAR) which has become the standard of care for patients with suitable anatomy, as EVAR has substantially improved early survival compared with open repair (23, 24). However, prophylactic treatment of asymptomatic AA does not come without risks. Despite a perioperative survival advantage of EVAR (1.1%) compared with open repair (4.4%) in infrarenal AAA, this advantage dissipates over time alongside high rates of reinterventions, concisely revealing some of the current challenges in aortic repair (25–27). These risks highlight the challenges for clinicians regarding a timely provision of elective surgical indication, to prevent the high mortality rate of 85% following rupture (28, 29).

These limitations emphasize the need for additional and novel imaging techniques with increased accuracy for rupture risk assessment, that will allow for a more patient centered approach for elective repair. This review article will highlight the development of novel clinical imaging techniques based on molecular pathophysiological insights of AA development, within the pre-clinical and clinical settings, targeting aneurysm-specific biomarkers for better risk stratification and disease monitoring.

## The State of the Art in AA Imaging

Commonly used imaging techniques for monitoring AAs include ultrasound scanning (US), computed tomography (CT) and magnetic resonance imaging (MRI), all mainly aimed at determining the aortic diameter or volume. Although the diameter is a quick and relatively easy parameter to determine, its use is limited as it can give inaccurate measure in asymmetric aneurysms. For this reason, new imaging parameters such as aneurysm volume and aortic size index (ratio of aortic diameter and body surface area) are being explored within these existing techniques (30–33). US uses high-frequency sounds waves and is an effective technique as it is time- and cost-efficient and allows for real-time imaging of the aorta without the use of radiation. Additional novel techniques such as contrast-enhancement using microbubbles makes US even useful for assessment of the microvascular anatomy of the aorta, providing valuable physiological information due to the physical and safety profiles of these administered microbubbles (34). Nevertheless, the interpretation of US images is user-dependent and provides relatively less detail compared to other imaging techniques in routine imaging procedures.

Computed Tomography Angiography (CTA) can provide full 3D reconstructions of the aorta (35). While non-contrast images allow for detection of calcification and previously applied surgical graft material (35), iodinated contrast agents are used to enhance bloodpool density and visualize arterial lumen. In addition, electrocardiogram (ECG)-gating of the CTA has the ability to correct for cardiac motion, providing increased image quality. Despite its wide applicability, it should be noted that CTA has an intrinsic relative disadvantage of using ionizing radiation, making it a less attractive solution alongside a growing awareness regarding the possible health risks (36). Nevertheless, this radiation dose has vastly decreased over the years, only producing a dosage of 10 milli-Gray in adults (37).

Moreover, magnetic resonance imaging (MRI) has also been used for 3D visualization of the aorta. This technique essentially does not require a contrast agent. However, contrast enhanced magnetic resonance angiography (MRA; with gadolinium) allows for additional imaging features and can provide useful information for the development of an AA such as 4D phase contrast (38, 39). This is relevant as the flow pattern and amount of flow in the false lumen is related to aortic expansion, and leading to secondary aneurysmal disease following the dissection (40). Nevertheless, MRI is a time-consuming and expensive diagnostic technique, which makes its current use limited in the contribution of routine preoperative and postoperative risk assessment of aneurysmal disease.

With our growing understanding of the pathophysiology and molecular mechanisms underlying aneurysmal disease, novel targets for molecular imaging have become available.

## Biology of Aortic Aneurysm Development

**Figure 1:**
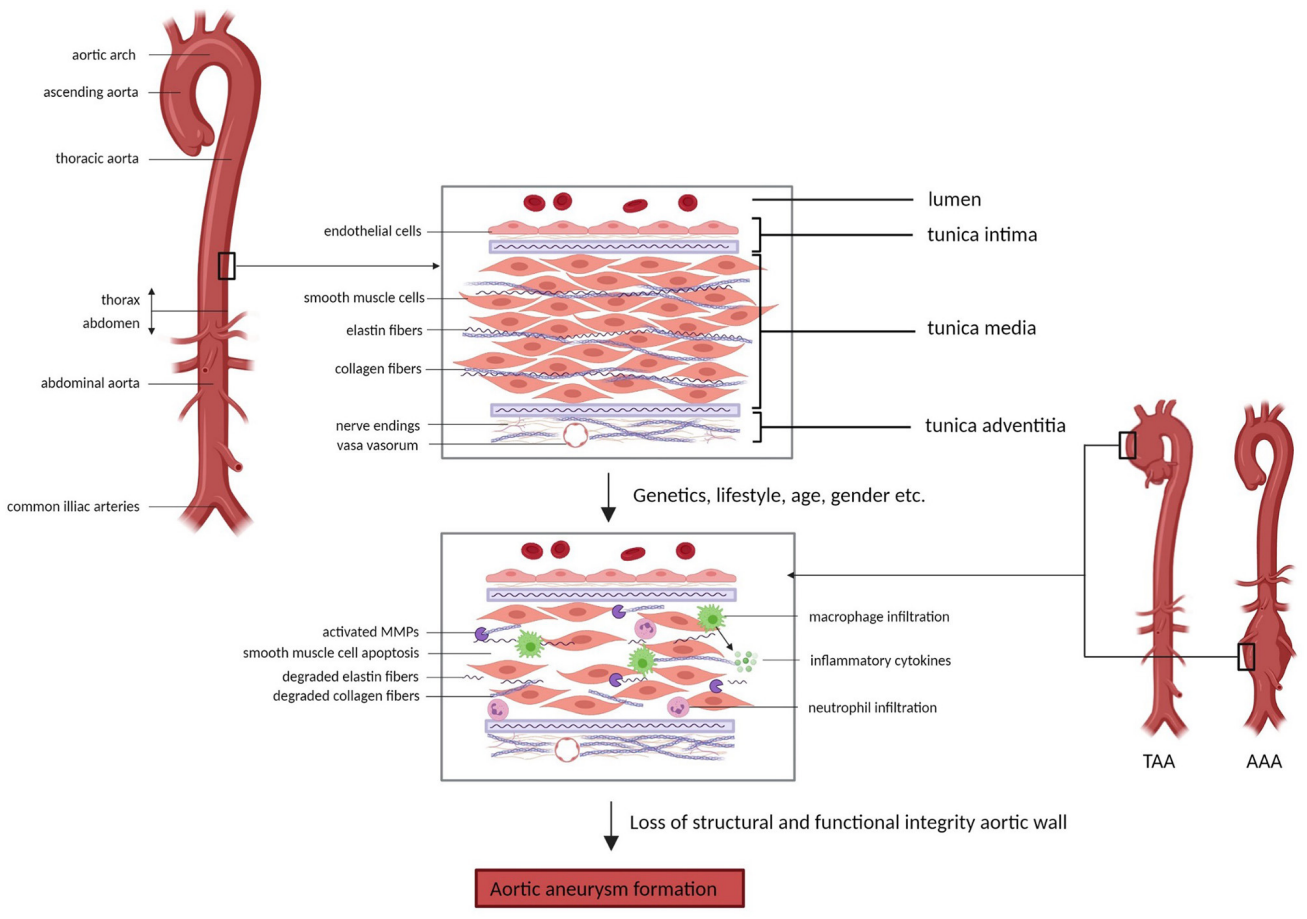
Aortic wall changes in aortic aneurysm formation. The aortic wall comprises three layers; the inner tunica intima, the middle tunica media and the outer tunica adventitia, which contain different cell types (e.g. endothelial cells and smooth muscle cells) and structures (e.g. elastin fibers and collagen fibers) that are essential in the maintenance of the structural integrity and function of the aorta. Factors such as genetics, lifestyle, age and gender can contribute to impairment of the structure and function of the aortic wall, eventually causing aortic aneurysm formation. Underlying processes involved in aneurysm development include smooth muscle cell apoptosis, degradation of extracellular matrix components and inflammation. *AAA* = *abdominal aortic aneurysm, TAA* = *thoracic aortic aneurysm, MMPs* = *matrix metalloproteinases* (Picture created with BioRender.com).

The aorta distributes oxygenated blood from the lungs to the systemic circulation. In order to facilitate the propulsion of blood from the heart to peripheral organs, the aorta possesses a high level of elasticity (41). Like other arteries, the vessel wall of the aorta is composed of three layers; the inner tunica intima, consisting of a basement membrane lined with endothelial cells (ECs), the middle tunica media, consisting of smooth muscle cells (SMCs) producing extracellular matrix (ECM) proteins, and the outer tunica adventitia, a connective tissue layer containing small vessels (vasa vasorum) and nerves (Figure 1) (42, 43). The ECM, present in all layers of the vessel wall, is a highly structured network containing elastin, collagen and proteoglycans and is essential for tensile strength and flexibility of the vessel wall. In pathological conditions such as AA formation, the structural and functional integrity of the vessel wall is compromised due to degeneration of the aortic media, characterized by loss of SMCs and aberrant synthesis, deposition or degradation of ECM proteins (42, 44).

Elastin and collagen are the main ECM proteins involved in pathological remodeling of the aortic wall upon aneurysm formation. Elastin is the main component of elastic fibers, which are organized into fenestrated sheets, or elastic laminae (43). Elastic laminae are situated between SMCs in the aortic media and provide elasticity to the aortic wall, allowing the aorta to expand and relax with each cardiac cycle (45, 46). Fibrillar collagens (predominantly type I and III) surround the SMCs and elastic laminae and provide structure and tensile strength to the aorta (47). Additionally, elastin and collagen fibers regulate SMC adhesion, proliferation and migration by interaction through integrins and other proteins (48, 49). Degradation or disorganization of elastin and collagen fibers therefore compromises arterial structure and function and has been shown to play an important role in aneurysm formation (50, 51). The degradation of elastin and collagen is regulated by matrix metalloproteinases (MMPs) and tissue inhibitors of matrix metalloproteinases (TIMPs) (52). Imbalance between the activity and presence of these MMPs and their inhibitors results in maladaptive remodeling of the ECM in the aortic wall. Normally, MMPs are produced by ECs, SMCs and fibroblasts and contribute to physiological remodeling of the aorta (53). Especially in AAAs, aneurysm formation involves a pronounced inflammatory component, with local infiltration of immune cells (mainly macrophages, T-cells and B-cells) into the aneurysmal aortic wall (54). Inflammatory cytokines (i.e., TGF-β, IL-1β, IL-6) trigger increased MMP production by SMCs and additionally, MMPs are produced by immune cells present in the aorta (55). Furthermore, circulating elastin fragments resulting from elastin degradation are able to trigger an inflammatory response, presumably through binding to macrophage surface receptors, which then causes further elastin degradation through MMP activation (43, 56). A large set of MMPs has been shown to be upregulated in the aneurysmal aorta, including MMP-1, MMP-2, MMP-3, MMP-9, MMP-12, MMP-13 and MMP-14 (57, 58). MMP-2, predominantly produced by SMCs, and MMP-9, predominantly produced by macrophages, were found to be the main MMPs involved in AAA development, causing ECM remodeling, apoptosis of SMCs and further inflammation (59, 60). These events compromise aortic wall integrity and consequently, prolong cyclic strain and increase wall tension resulting in aortic dilation.

Despite similarities in macroscopic appearance, TAAs and AAAs hold distinctly different underlying pathophysiological mechanisms. This can partly be attributed to the different embryological origin of cells from the thoracic and abdominal aorta (61). Additionally, there are structural differences, with the abdominal aorta containing less fibromuscular layers and vasa vasorum compared to the thoracic aorta (62). Lastly, the underlying causes of aneurysm formation differ between the thoracic and the abdominal aorta, where genetic deficiencies play a large role in TAA development and a more multifactorial process underlies AAA development with lifestyle as a large contributor. Accordingly, different targets for diagnostics and therapy might be of interest between TAAs and AAAs. Therefore, it is important to discriminate between molecular mechanisms of AA development in the thoracic vs. the abdominal part of the aorta.

### Genetics and Molecular Mechanism of TAA Development

TAAs are often syndromic and are generally caused by variants in genes involved in ECM integrity and structure (e.g. *FBN1* and *EFEMP2*), transforming growth factor β (TGF-β) signaling (e.g. *SMAD3* and *TGFBR1/2*) and cytoskeleton maintenance and motility (e.g. *ACTA2* and *MYH11*)(13). Heterozygous mutations in the gene for ECM protein Fibrillin-1, *FBN1*, cause Marfan syndrome (MFS1; MIM 154700) in patients, an autosomal dominant disorder with wide clinical variability affecting the cardiovascular, ocular and skeletal systems (63). Homozygous or compound heterozygous mutations in the gene for ECM protein Fibulin-4, *EFEMP2*, cause cutis laxa syndrome (ARCL1B; MIM 614437) in patients, characterized by systemic connective tissue abnormalities, including loose skin, lung emphysema, bone fragility, vascular tortuosity and aneurysms (64). Mutations in the *ELN* gene, encoding for elastin, can also cause cutis laxa syndrome (ADCL1; MIM 123700) (65). These ECM proteins are involved in vascular maturation and maintenance of structural integrity and elasticity of the aortic wall. Fibrillin-1 and Fibulin-4 proteins form microfibrils in the ECM, where they associate with other ECM proteins such as elastin and are therefore essential for the structural integrity of the aortic wall. Both Fibrillin-1 and Fibulin-4 associate with latent TGF-β binding protein (LTBP) and are thereby involved in the regulated release of TGF-β from large latent complexes (LLCs) in which free TGF-β is bound to the ECM (66–68). Consequently, deficiency in these proteins results in increased release of active TGF-β and dysregulated TGF-β signaling (69, 70). Downstream effects are increased activity of MMPs, elastin degradation, deposition of ECM proteins and aberrant cytoskeleton fibers. This causes increased stiffness of the aortic wall, making it less resilient to mechanical stress which promotes aneurysm formation.

*TGFBR1/2* and *SMAD2/3* encode proteins involved in TGF-β signaling and mutations in these genes cause Loeys-Dietz syndrome (LDS) in patients *(TGFBR1;* LDS1; MIM 609192*, TGFBR2;* LDS2; MIM 61068*, SMAD2;* LDS6; *MIM 619656, SMAD3;* LDS3 (AOS); MIM 613795). LDS is characterized by arterial aneurysms, dissections and cardiac abnormalities (71, 72). As *TGFBR1/2* and *SMAD2/3* are important regulators of the TGF-β pathway, deficiency of these proteins causes dysregulated downstream signaling of this pathway in LDS patients (72–74). Paradoxically, increased *pSMAD2* signal is detected in the aortic wall of *TGFBR1/2* and *SMAD3* patients, suggesting TGF-β pathway activation whereas pathway inhibition is expected (75, 76). This can be explained by the fact that upstream TGF-β signaling is activated, but downstream the signal is not transduced resulting in a discordance between up- and downstream TGF-β pathway activation (77). Aneurysm development in LDS patients is characterized by a pronounced inflammatory response, which is thought to be initiated by changes in aortic wall structure and subsequently triggers rapid progression of aneurysmal growth.

Mutations in *ACTA2, MYH11, MYLK* and *PRKG1* lead to non-syndromic familial aortic aneurysms (AAT1; MIM 607086) (76). The proteins encoded by these genes are involved in SMC contractility, therefore deficiency of these proteins functionally impairs contraction of the aortic wall. Additionally, increased TGF-β signaling was found in the aorta of patients, again emphasizing the role of this signaling pathway in aneurysm formation (78). Dysfunctional actin and myosin filament assembly impairs proper formation of fibronectin fibrils in the ECM. This further causes aberrant fibrillin-1 microfibril assembly, causing increased release of free TGF-β and promoting TGF-β signaling.

### Molecular Mechanism of AAA Development

As previously mentioned, AAA development is generally caused by a multifactorial process and cannot be attributed to a single cause. Risk factors for AAA development are smoking, advanced age, male gender, hypertension, and family history (79). Despite the important role of lifestyle in AAA development, a prior systematic review identified 33 single nucleotide polymorphisms (SNPs) associated with AAA diagnosis at genome-wide significance that were previously validated in multiple cohorts. However, the functional consequences of these SNPs on AAA growth still have to be established. Furthermore, since AAA primarily affects elderly men, the role of sex hormones are becoming of increased interest. Villard et al. have recently reported an association between increased progesterone levels and early AAA development within males, as well as higher circulating levels of estradiol and lower levels of testosterone in men with AAA (80). Similarly, in comparison to females before their menopause, the higher prevalence of cardiovascular disease in postmenopausal females has been found to be related to the diminishing levels of female sex hormones during menopause.

At the cellular level, hallmarks of AAA pathology include apoptosis of SMCs, ECM degeneration, inflammatory cell infiltration and oxidative stress (44). As described previously, these cellular processes cause a loss of structural integrity of the aortic wall, consequently leading to aortic dilation and eventually rupture. An imbalance in MMP activity causes ECM degeneration, triggering reduced levels of elastin, collagen and glycosaminoglycans in the aortic wall. AAA formation is characterized by a pronounced inflammatory response and therefore macrophages and neutrophils, as well as inflammatory factors such as TNF-α, TGF-β and interleukins, are often present in the aneurysmal aorta (81).

Thus, several of these previously described processes are characteristic for TAA and AAA development and progression, respectively, and therefore are interesting targets for diagnosis and monitoring of disease in patients. As AAAs are often attributed to a convergence of multifactorial genetic causes combined with external factors, it is a lot more challenging to identify molecular mechanisms at play in each individual patient compared to TAA patients for which the single genetic cause and thus the associated molecular mechanism can be used as a target for molecular imaging. However, the majority of AA cases are abdominal (82), so there is an even bigger need to find suitable imaging techniques for this type of AA. Not every imaging technique might be applicable to monitor both TAAs and AAAs, and effectiveness may vary dependent on the different mechanisms underlying AA formation and development. This emphasizes the importance of understanding the molecular mechanisms governing AA, which potentially can be elucidated with pre-clinical research in animal models.

## Mouse Models for Aortic Aneurysm

Aneurysmal mouse models are used to test the effectivity of imaging techniques as disease development and progression are predictable and can be closely monitored. Mice have a fast reproduction rate, a short lifespan and are genetically similar to humans, and are therefore considered to be of value in medical research (83). Moreover, mice can be bred in an isogenic background, by which the gene or process of interest can be studied, not affected by genetic heterogeneity. Many different murine models for AA have been created in which we can distinguish between TAA and AAA models, either genetically or chemically induced, or a combination of both. As the pathophysiology is unique for TAA and AAA and as AA development is a multigenetic and multifactorial process, these models subsequently elucidate individual mechanisms involved in aneurysm formation.

### TAA Mouse Models

**Figure 2:**
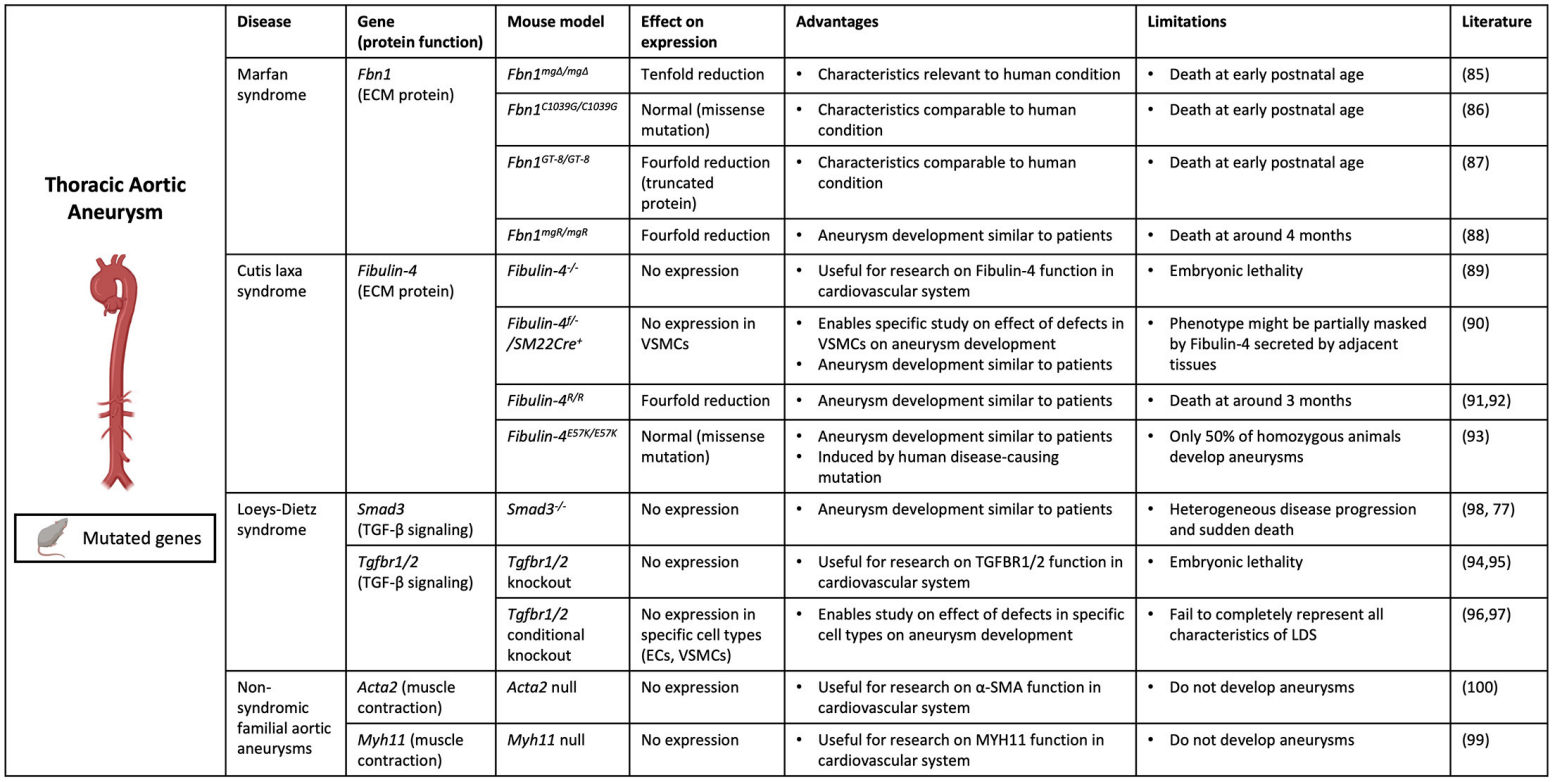
Contemporary murine models for TAA. Thoracic aortic aneurysm can be modeled in mice through genetic modification of specific genes (*Fbn1, Efemp2 (Fibulin-4), Smad3, Tgbr1/2, Acta2* and *Myh11*) involved in aneurysm formation. (Picture created with BioRender.com).

Aneurysms in the thoracic part of the aorta typically result from degeneration of the aortic media with apoptosis of SMCs and elastic fiber degradation. Several genetic deficiencies resulting in TAA formation in patients, as described previously, have been mimicked in mouse models to study the underlying pathophysiological pathways (84) (Figure 2).

Several mouse models were created to mimic Marfan syndrome, in which patients develop TAAs due to Fibrillin-1 deficiency. Marfan syndrome was first mimicked in *Fbn1*^*mgΔ*/*mgΔ*^ mice, with an in-frame deletion of exons 19–24 in the *Fbn1* gene causing severe (ten-fold) reduction of *Fbn1* expression (85). These mice die at an early postnatal age of 3 weeks due to cardiovascular complications, which complicates the study of disease progression. Additional murine models for Marfan syndrome include the *Fbn1*^*C*1039*G*/*C*1039*G*^ model, in which mice have a missense mutation causing normal *Fbn1* expression but aberrant fiber formation (86), and the *Fbn1*^*GT*−8/*GT*−8^ model, where Fibrillin-1 is truncated and has four-fold reduced expression (87). However, these mice also die at a postnatal age when harboring the homozygous mutation. To overcome this, mice with a hypomorphic *Fbn1* mutation (mgR), causing a four-fold reduction in normal Fibrillin-1 expression, were generated (88). These *Fbn1*^*mgR*/*mgR*^ mice develop aneurysms similarly to human Marfan patients, showing vascular inflammation and elastin degradation in the aortic wall, and die at around 4 months as a result of aortic dissection. This makes this model not only useful for investigating underlying molecular mechanisms of disease, but also suitable for testing imaging techniques targeting major processes involved in aneurysm development in Marfan patients.

Additionally, to study the underlying mechanism of aneurysm formation in cutis laxa syndrome caused by Fibulin-4 deficiency, several mouse models have been developed. Complete knockout of the *Efemp2* gene, the gene encoding Fibulin-4, appeared to cause perinatal lethality in mice (89). Therefore, an SMC specific knockout of *Fibulin-4* (*Fibulin-4*^*f*/−^*/SM22Cre*^+^) was generated, causing AA formation in the ascending aorta (90). Additionally, mutant mice were developed with a four-fold decreased expression of *Fibulin-4* (*Fibulin-4*^*R*/*R*^) (91). *Fibulin-4*^*R*/*R*^ mice show dilation and tortuosity of the ascending aorta and die of aortic dissection at an early age of 3 months. Aneurysm formation in *Fibulin-4*^*R*/*R*^ mice is highly similar to that in patients at a macroscopic and molecular level. *Fibulin-4*^*R*/*R*^ aneurysmal aortas show elastin fragmentation and increased deposition of ECM, as well as increased TGF-β signaling and mitochondrial dysfunction (91, 92). This model has proven to be very valuable for studying underlying molecular mechanisms of aneurysm formation, but also for pre-clinical testing of imaging techniques targeting these molecular processes. Lastly, a knock-in mouse model was created carrying the human disease-causing E57K mutation in the *Fibulin-4* gene, causing normal protein expression but reduced ECM assembly (93). *Fibulin-4*^*E*57*K*/*E*57*K*^ mice show characteristics similar to human patients and develop ascending aortic aneurysms, which makes this model useful for studying underlying molecular mechanisms of disease and potentially also pre-clinical testing of imaging techniques targeting these processes.

Furthermore, several mouse models were created to mimic LDS, which is caused by mutations in *TGFBR1/2* and *SMAD3* genes in patients. Both *Tgfbr1* and *Tgfbr2* deletion cause embryonic lethality in mice due to severe defects in vascular development (94, 95). Conditional knockouts of *Tgfbr1* and *Tgfbr2* in ECs and SMCs show several symptoms of LDS but fail to completely represent all characteristics (96, 97). *Smad3* knockout mice, although in isogenic background, show similar heterogeneity in disease development to patients and die suddenly from TAA formation (98). The aortic wall of these mice shows elastin disruption and a pronounced infiltration of inflammatory cells with MMP activity, which promotes further deterioration of the aortic wall and could therefore explain the rapid disease progression (77).

Lastly, mouse models were developed to represent disease development as seen in familial TAA in patients with *ACTA2* and *MYH11* mutations. However, despite causing cardiovascular abnormalities, complete loss of ACTA2 and MYH11 protein expression does not cause aneurysm formation in mice which makes these models unsuitable for studying *ACTA2* and *MYH11* induced aneurysmal disease development (99, 100). However, Angiotensin II infusion of *Acta2*^−/−^ mice did result in enhanced dilation of the thoracic aorta, demonstrating that deficiency of ACTA2 promotes aneurysm formation in combination with chemical induction (101).

### AAA Mouse Models

**Figure 3:**
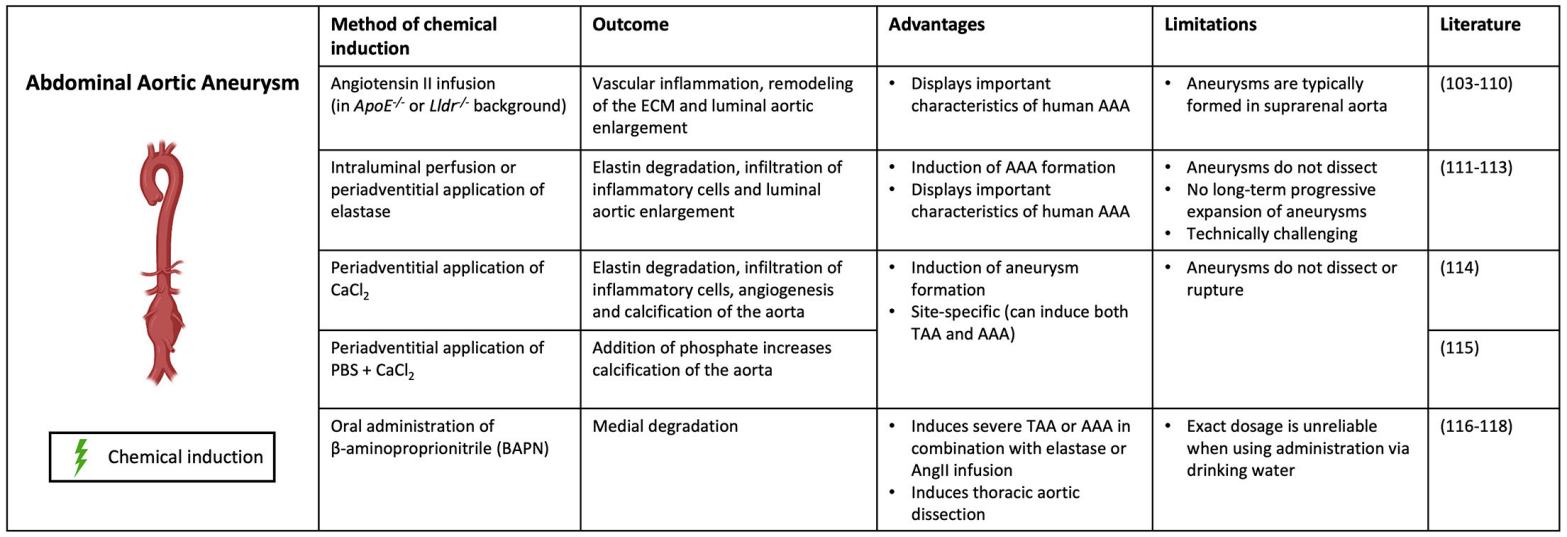
Contemporary murine models for AAA. Abdominal aortic aneurysm can be modeled through chemical induction using Angiotensin II (in *ApoE*^−/−^ and *Lldr*^−/−^ mice), elastase, calcium chloride [CaCl_2_, with/without phosphate (PBS)] and β-aminoproprionitrile (BAPN). (Picture created with BioRender.com).

Several mouse models of AAAs have been developed that use a combination of genetic manipulation and chemical induction for reproducing aneurysmal disease development (Figure 3). Hyperlipidemic apolipoprotein E deficient (*ApoE*^−/−^) or low-density lipoprotein receptor deficient (*Lldr*^−/−^) mice were initially used in research on atherosclerosis but were found to develop AAAs when being fed a high-fat diet (102). This model represents similar characteristics of vascular inflammation, atherosclerosis and AA formation caused by obesity in humans.

The most common method to induce AAA in *ApoE*^−/−^ or *Lldr*^−/−^ mice is infusion of Angiotensin II (AngII), a hormone which increases blood pressure by promoting vasoconstriction and aldosterone secretion (103, 104). However, not the increase in blood pressure, but rather the induction of inflammation in the aorta is what causes aneurysm formation in the AngII model (105). While the AngII–infused ApoE–deficient mouse is one of the most widely used murine models for human AAA due to vascular inflammation, remodeling of the ECM, and activation of SMCs, its limitations are well recognized (106). These mice typically form aneurysms in the suprarenal aorta, whereas human AAAs are most commonly infrarenal (107). Differences in elastin and collagen composition between locations might impact mechanical properties of the aortic wall (108). In addition, the animals tend to form aneurysms as a consequence of hypertension-induced aortic dissection and intramural hematoma, whereas the vast majority of AAA disease in humans is not secondary to these diseases, but a consequence of smoking and atherosclerosis (109). AngII induction has also been shown to cause AAA formation in wildtype C57BL/6 mice, but the incidence is much lower than in hyperlipidemic models (110).

Another commonly used method to chemically induce AAA formation uses elastase. In this model, the aorta is exposed to porcine pancreatic elastase by either intraluminal perfusion or periadventitial application (102, 111). Elastase penetrates into the aortic medial layer, where it causes elastin degradation and subsequent infiltration of inflammatory cells and luminal aortic enlargement (112). Despite the fact that this technique is able to properly induce AAA formation in mice, these aneurysms do not dissect. Additionally, these aneurysms show no long-term progressive expansion as they usually stabilize after 2 to 3 weeks (111, 113). Furthermore, the technique is technically challenging making it a less preferable model for AAA induction.

Application of calcium chloride (CaCl_2_) or calcium phosphate (CaCl_2_ and PBS) to the adventitia of the aorta is another method to chemically induce AAA formation. This model induces elastin degradation, infiltration of inflammatory cells, angiogenesis and calcification of the aorta, subsequently resulting in AAA formation (114). The addition of phosphate increases calcification of the aorta (115). This model is site-specific and can therefore be used to induce both AAAs and TAAs, which is advantageous compared to the other methods. However, this model does not result in dissection or rupture of the aorta, making it less representative for human AA formation.

Lastly, oral administration of the lysyl oxidase (LOX) inhibitor β-aminopropionitrile (BAPN) is a method to chemically induce both AAA and TAA formation in mice. BAPN causes medial disruption by blocking crosslinking of elastin and collagen, resulting in reduced stability of the ECM (116). It has been used in combination with elastase or AngII infusion to induce severe AAA or TAA, promoting aortic rupture and dissection respectively (117). When administered individually, BAPN has been shown to induce thoracic aortic dissection (118). The fact that BAPN promotes dissection and rupture of the aorta, thereby representing important features of human AA formation, makes this a clinically relevant model (102).

These mouse models represent several important characteristics of human AAA development and therefore form interesting models for testing diagnostic and therapeutic markers. However, these methods are considered to be quite invasive to induce AAA formation and none of these models fully represent the natural progression of AAA growth in human patients. A complicating factor of pre-clinical testing in mice is that molecular pathways that are changed in these mice could be different, or not at all affected in patients, therefore impairing translatability. The fact that aneurysm development and progression is much faster in mice compared to the chronic AAA development seen in patients contributes to this concern. Difficulties in establishing a representative mouse model that mimics human AAA are attributed to the fact that in AAAs there is not one single cause of disease. Many risk factors such as smoking, age, weight and genetic variances are encountered during a long period of time and are hard to replicate in a model organism. Moreover, even a combination of all risk factors does not guarantee growth of an AAA. On the contrary, since TAAs are mostly caused by a single genetic defect, mouse models have been successfully created that represent important characteristics of TAA development in human patients. This makes these models more suitable for pre-clinical testing of imaging techniques targeting underlying molecular mechanisms of TAA development, since these are similar in mice compared to human patients.

Despite the development of several murine models that quite accurately mimic human AA disease at a macroscopic and molecular level, the use of murine models *per se* presents with limitations concerning translatability. The human and murine genome are largely similar, but not all physiological and pathological processes can be identically reproduced in mice. For example, distinct differences were found in response to inflammatory trauma in terms of gene expression (119). Considering the important role of inflammation in AA disease, these findings imply that we should be mindful with the extrapolation of pre-clinical findings to the clinical setting. Despite these facts, murine models have been largely applied and proven useful for pre-clinical testing of imaging techniques to diagnose and monitor AA development and progression.

## Pre-Clinical Aortic Aneurysm Imaging

At present, several non-invasive high-resolution imaging techniques, including US, micro computed tomography (μCT), MRI and photo-acoustic imaging (PAI), are commonly applied to monitor AA development and progression in mice (116). The approach of US is similar in pre-clinical and clinical applications, besides the use of a higher frequency when imaging mice, producing images with higher spatial resolution (120–122). US has different modalities which can be applied to obtain more detailed information on the vasculature such as Doppler US examination to measure blood flow velocity, M-mode US examination for quantifying circumferential strain, and volumetric US examination to visualize complex lesions. Another pre-clinical imaging technique is small animal μCT, which produces high-resolution three-dimensional images of the aorta by using a vascular contrast agent that allows for accurate measurement of aortic volume (123, 124). Furthermore, high-field MRI using small bore systems, especially in combination with contrast agents targeting specific markers, has been proven valuable in pre-clinical imaging of AA disease in murine models (125, 126). Lastly, PAI is a novel method, which relies on the detection of ultrasound waves generated by the interaction of light pulses with tissue and molecules and their subsequent thermo-elastic expansion. In other words, light pulses are converted to ultrasound waves, enabling deeper tissue penetration compared with light detection. Endogenous contrast can be generated by the presence of light absorbers like hemoglobin or melanin while exogenous contrast can be generated by probes which absorb light in the NIR (near infrared region) such as fluorescent proteins, dyes and nanoparticles. PAI, for example, allows the simultaneous visualization of changes in the oxygenation of the blood (e.g., due to local hypoxia) and precise localization of specific targeted probes, thereby providing anatomical, functional and molecular information. Studies already successfully applied PAI to image atherosclerotic tissue, since this technique allows the detection of lipid distribution, and is also suggested to be suitable for imaging aneurysms (127).

**Figure 4:**
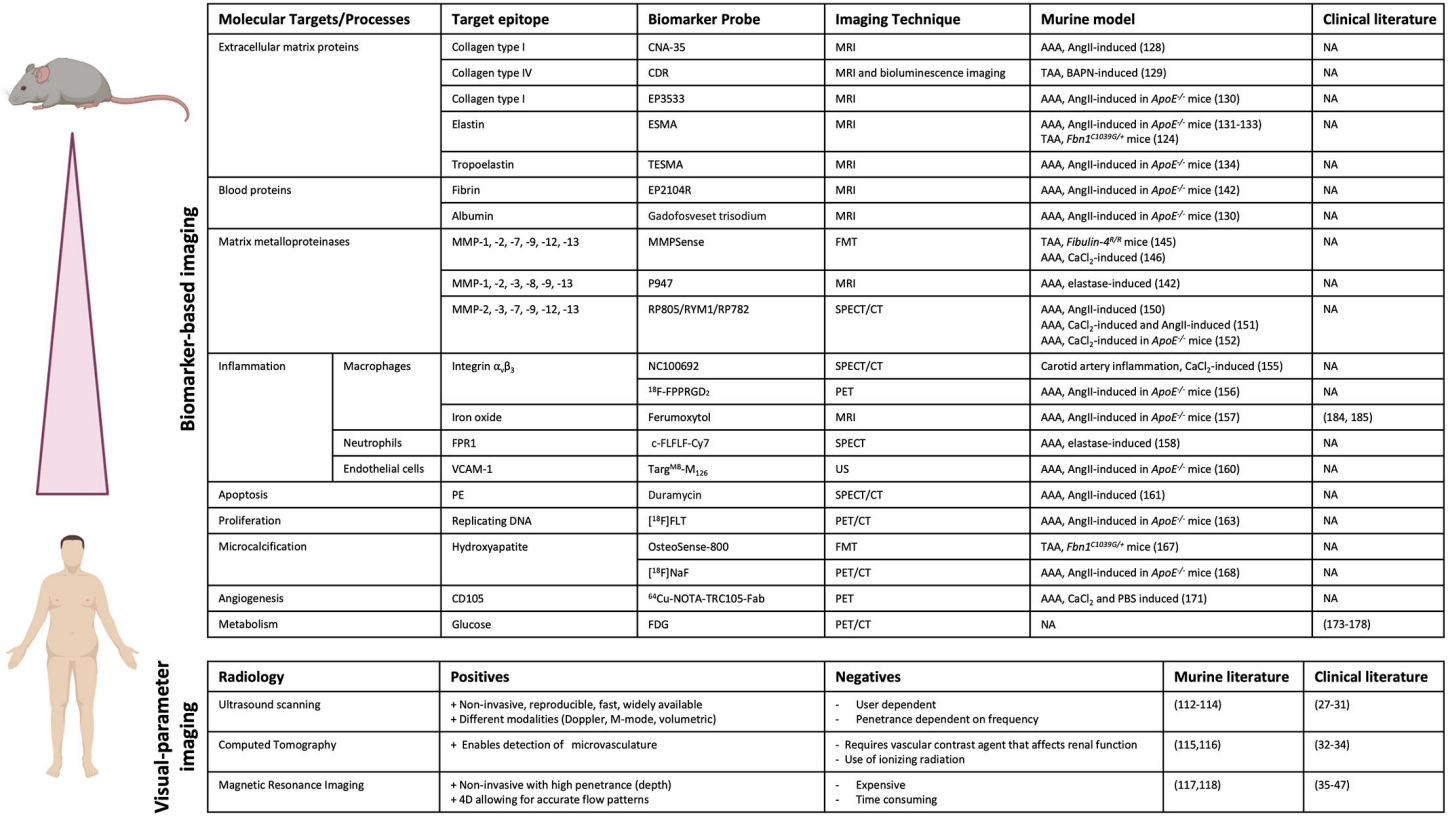
Pre-clinical and clinical imaging techniques for AA. Overview of biomarker-based imaging techniques and visual parameter-based imaging techniques that have been tested in pre-clinical trials using murine models and/or in clinical trials with patients. (Picture created with BioRender.com).

Currently, novel imaging techniques are being explored that apply molecular targeting of specific underlying pathways involved in AA formation and progression. Several of the previously mentioned mouse models have been used in pre-clinical trials to test these novel imaging techniques. Molecular targets that are under investigation for imaging applications with respect to AA formation include ECM proteins, MMPs, immune cells and SMC phenotype (Figure 4).

### Detection of Extracellular Matrix Proteins in TAA and AAA

As previously described, degradation of ECM proteins, like collagen, is an important process involved in AA development and therefore forms an interesting target for diagnostic imaging to monitor disease progression. A method to image collagen degradation in mouse aorta is through paramagnetic/fluorescent nanoparticles containing a collagen-I-binding protein (CNA-35) (128). Following intravenous injection of the nanoparticles, the primary fibrotic response in the aortic wall can be visualized with high-resolution, multisequence MRI. The CNA-35 probe significantly improved MR signal in AngII-induced aneurysmal mice, but its predictability of AAA development and progression remains to be proven. In a different study, a collagen-IV-targeted MRI/fluorescence dual probe (CDR) was developed, which enabled early detection of collagen type IV exposure in the aortic wall and was shown to form a suitable marker for early prediction of rupture risk in a BAPN-induced TAA model (129). The percentage normalized signal enhancement (NSE) was significantly higher in animals with ruptured TAAs compared to animals with stable, unruptured TAAs and NSE was negatively correlated with the time until rupture after BAPN administration. Additionally, a gadolinium-based probe that specifically binds to type I collagen (EP-3533) was created to enhance MR images. Use of this probe enabled differentiation between collagen-rich and collagen-poor aneurysms, of which the latter is more prone to rupture, in AngII-infused *ApoE*^−/−^ mice (130). Since these probes have been tested in chemically induced models of AAA, future clinical trials will have to prove viability of this concept in human disease progression and will have to subsequently demonstrate its added value to prediction of rupture risk.

Similar to collagen, elastin is a key structural protein for integrity of the aortic wall and distinctive for AA development. Therefore, targeting elastin in MRI imaging could be useful for AA risk analysis. A gadolinium-based, elastin-specific magnetic resonance molecular imaging agent (Gd-ESMA) was used to image AAA in *ApoE*^−/−^ mice infused with AngII (131). ESMA exhibits preferential binding to elastin molecules and is labeled with gadolinium as a contrast agent. Gd-ESMA-enhanced MR images were compared to *ex vivo* area measurements, which confirmed the correlation between enhanced MRI signal and presence of elastin in the aortic wall. Additionally, Gd-ESMA was able to accurately detect a decrease in elastin content in the aortic wall of Marfan mice (*Fbn1*^*C*1039*G*/+^) compared to wild-type controls (132). When comparing Gd-ESMA to a non-specific contrast reagent (Gd-DTPA), which shows no selectivity toward elastin, it was apparent that Gd-DTPA did not result in an enhanced image of the aneurysmal wall, whilst Gd-ESMA clearly improved imaging by specifically targeting elastin (131). Gd-ESMA was also used to study the therapeutic effect of IL−1β neutralization on AAA progression in AngII-infused *ApoE*^−/−^ mice by monitoring elastin degradation in the aortic wall (133). Besides early *in vivo* evaluation and quantification of AAAs, the probe enabled monitoring of the response to anti-inflammatory treatment. Gd-ESMA enables the prediction of AAA rupture with a sensitivity of 80% and a specificity of 78%, further suggesting that elastin is a useful marker for the prediction of rupture risk (134). Additionally, tropoelastin, a monomeric precursor of cross-linked elastin, was also shown to be increased upon AAA formation and to correlate with disease progression. Use of a gadolinium-based tropoelastin-specific MRI contrast agent (Gd-TESMA) to enhance MRI images, based on a peptide that selectively binds to tropoelastin, enabled identification of dysfunctional ECM remodeling in aneurysmal tissue of AngII-infused *ApoE*^−/−^ mice (135). However, the predictive value of Gd-TESMA enhanced imaging concerning AAA progression and rupture still has to be proven. Thus, monitoring elastin degradation or tropoelastin accumulation using MRI in combination with probes that specifically target these molecules could provide a useful tool to monitor aneurysm formation and potentially assess the risk of rupture.

Additional ECM proteins that have been shown to be upregulated in AAA tissue, and that might serve as suitable targets for molecular imaging, are thrombospondin, fibronectin and periostin (136, 137). Thrombospondin-1 is a secreted adhesive glycoprotein that mediates cell-to-matrix interactions by binding to ECM proteins such as collagen, fibrinogen and fibronectin. Periostin is an ECM protein that binds to integrin molecules on cell surfaces and its upregulation has been shown to correlate to AAA progression and inflammation, possibly making it an interesting target for monitoring disease progression (138).

### Detection of Blood Proteins in AAA

Fibrin is a fibrous protein involved in blood clotting and is associated with formation of focal hematoma in aortic wall dissection and the development of larger thrombi during AAA progression (139, 140). Increased turnover of the fibrin protein was recently linked to stimulation of inflammatory pathways (139–141). This marker is especially important as thrombus growth is increasingly seen as a predictor of rupture in AA (142). Molecular MRI with a fibrin-specific molecular probe (EP2104R) was tested in AngII-infused *ApoE*^−/−^ mice (143). This method allowed for detection of aortic dissection even before visible aortic dilation, by visualizing hematoma formation. Moreover, no relevant expression of collagen or elastin was measured on histological sections at this early time point in disease development, meaning no structural changes in the ECM had taken place yet (143). Early phase thrombus also resulted in an enhanced MR signal with the fibrin-specific probe. In later stages the signal decreased, but at this time expression of collagen and elastin in the thrombus increased, allowing for early- and late phase thrombus distinction. Therefore, this probe could contribute to more accurate AAA rupture risk prediction in patients and is especially interesting since it has already been successfully clinically tested (144).

A different study used detection of albumin, one of the major plasma proteins in the blood, to visualize changes in vascular permeability that are observed in AAA formation. Gadofosveset trisodium, a gadolinium-containing MRI probe that binds to albumin, enhanced *in vivo* MR signal in AngII-infused *ApoE*^−/−^ mice and was able to predict AAA rupture with 100% sensitivity and 86% specificity in these mice (130). Therefore, this albumin-targeting probe shows great potential for AAA risk prediction in a clinical setting, especially since it has already been clinically approved.

An additional blood protein that is upregulated in AAA and that might serve as a suitable target for molecular imaging is Factor XII (FXII). FXII is involved in thrombus formation and its expression and activity correlate with AAA size. Furthermore, inhibition of FXIIa (the active form of FXII) activity as well as FXII depletion inhibit dilation of the suprarenal aorta in AngII-infused *ApoE*^−/−^ mice. However, while FXII is found in increased amounts in the serum of patients with expanding AAA, the combination of FXII with aneurysm diameter had limited predictive effect on aneurysm progression vs. diameter alone.

### Detection of Matrix Metalloproteinases in TAA and AAA

As previously mentioned, activity and presence of MMPs is increased in AAs, resulting in degradation of the ECM in the aortic wall. Therefore, MMPs form a useful target for diagnostic imaging to visualize AA development and progression. The protease-activatable near-infrared fluorescence (NIRF) probe MMPSense 680 targets MMP-1, MMP-2, MMP-7, MMP-9, MMP-12 and MMP-13 activity, enabling *in vivo* visualization of MMP activation using fluorescence mediated tomography (FMT)-CT imaging. This probe was able to detect increased MMP activity even before aneurysms had formed in aortas of *Fibulin-4*^*R*/*R*^ mice, proving its value as a predictive biomarker for TAA formation (145). The MMPSense probe also showed promising results in a CaCl_2_-induced AAA mouse model, where fluorescent signal correlated to AAA growth inhibition by doxycycline treatment (146). In a different study, a contrast agent named P947 was developed that targets a wide range of MMPs (147, 148). In elastase-infused rats, the P947 contrast agent resulted in a significantly enhanced MR signal (149). The predictive value of this marker targeted by this contrast agent still has to be assessed. A limitation is the possibility that elastase-induction itself may affect the activity of MMPs and thus enhance the image in this model. Hence, exploring this molecular probe in other mouse models, such as the previously mentioned genetic TAA mouse models that do not require chemical induction for aneurysm formation, could be useful to exclude false-positive image enhancement due to elastase administration. Another technique to target MMPs is Single Photon Emission Computed Tomography (SPECT). SPECT uses a radioactive tracer in combination with CT. In AngII-infused mice, MMPs were targeted using RP805, a technetium-99m-labeled (^99m^Tc) MMP-specific tracer (150). This study showed a significant increase in uptake of RP805 in mice that later developed rupture or AAA, showing its predictive value (150). The ^99m^Tc-RYM1 tracer, based on the MMP inhibitor RYM, showed favorable pharmacokinetics compared to RP805 and was able to specifically detect MMP activity in both CaCl_2_ and AngII-infused mice (151). ^99m^Tc-RYM1 signal was significantly higher in the AAA group. Similarly, RP782, a indium-111m-labeled (^111^In) MMP tracer, was used in CaCl_2_-induced *ApoE*^−/−^ mice to enhance the SPECT image (152). Increased RP782 uptake correlated with later aneurysm expansion, suggesting that RP872 enhanced imaging can predict future aneurysmal growth. Thus, several studies suggest that MMP targeted molecular imaging is a useful tool for more accurate prediction of aneurysm growth and rupture. These results are particularly relevant due to the wide availability of CT equipment in hospitals and considering the speed and cost-effectiveness of SPECT imaging.

### Detection of Immune Cells in AAA

Besides ECM proteins, immune cells and inflammatory cytokines are also known to play a key role in aneurysm progression. Immune cells accumulate in the aortic wall at the site of aneurysm formation and produce (pro-inflammatory) cytokines and proteases, which stimulate breakdown of the SMC layer (153). Macrophages are one of the primary immune cells infiltrating into the aortic wall upon aneurysm formation, making them a relevant target for diagnostic imaging. Additionally, the number of macrophages is increased in dissected areas, suggesting that the amount of macrophages is a predicting factor for aneurysm progression and rupture (154). Specific targeting of integrin α_v_β_3_, expressed on the cell-surface of monocytes and macrophages, using a ^99m^Tc-cyclic arginine-glycine-aspartate (RGD) peptide tracer (NC100692) for μSPECT/CT has been proven successful in detecting vascular inflammation in a CaCl_2_-infused murine model of carotid artery inflammation (155). This study did not yet investigate the predictability of aneurysm growth or rupture of this marker. The radiolabeled RGD positron emission tomography (PET) tracer ^18^F-FPPRGD_2_, which also targets integrin α_v_β_3_, was able to detect vascular inflammation in AngII-infused *ApoE*^−/−^ mice but did not correlate with aneurysm size (156). This suggests that integrin α_v_β_3_ might be less suitable as a target for predicting aneurysm growth and rupture. A different method to specifically target activated pro-inflammatory macrophages is by exploiting their uptake and retention of iron particles (157). MRI analysis targeting macrophages with an iron-oxide reagent (ferumoxytol) was performed in AngII-infused *ApoE*^−/−^ mice (134). The iron-oxide reagent could predict aneurysm rupture with a sensitivity of 80% and a specificity of 89%, in comparison to Gd-ESMA which showed a sensitivity of 80% and a specificity of 78%. The best result was obtained with a combination of the iron-oxide and elastin probes which could predict rupture with selectivity and specificity of 100% and 89%, respectively (134). Additionally, a different study combined the type I collagen-targeted gadolinium-based probe (EP−3533) and the iron oxide-based probe (ferumoxytol) to simultaneously image ECM remodeling and inflammation in aortas of AngII-infused *ApoE*^−/−^ mice (130). Combined use of these probes yielded a sensitivity of 80% and a specificity of 100% for AAA rupture prediction, which again was superior compared to the predictive abilities of the individual probes alone. These probes do not interfere with each other's visualization and quantification and their combined use greatly enhances the amount of information obtained from one single MRI scan. Thus, this dual-probe molecular MRI approach looks very promising and could have a lot of potential in clinical settings, especially since the iron-oxide agent is already being used in the clinic and the gadolinium-based agents are very similar to currently clinically applied contrast agents.

Infiltration and activation of neutrophils has also been shown to be involved in the inflammatory response in AAAs. Activated neutrophils can be targeted for *in vivo* imaging via the formyl peptide receptor 1 (FPR1), using the FPR1 ligand c-FLFLF labeled with either Cy7 to enable fluorescence imaging or ^99m^Tc to enable SPECT imaging (158). This probe enabled the detection of AAA development and progression in elastase-infused mice with both imaging techniques and shows potential for the prediction of aortic rupture.

Theranostics, the combination of therapeutics and diagnostics, is a novel technique that applies agents that can simultaneously be used for diagnostic imaging as well as therapeutic drug delivery. A molecule that has been targeted for theranostics is vascular cell adhesion molecule-1 (VCAM-1) which is present on inflamed ECs and mediates accumulation and adhesion of leukocytes. VCAM-1 is upregulated in AAA and has been shown to play a role in the inflammatory response in aneurysms (159). Microbubbles coated with an antibody directed toward VCAM-1 (Targ^MB^-M_126_) were applied for simultaneous ultrasound imaging and drug delivery of a microRNA (miR-126), which downregulates VCAM-1 expression, in AngII-infused *ApoE*^−/−^ mice (160). This novel theranostic application provides a functional contrast agent to assess the inflammatory state of the endothelium as well as targeted delivery of therapeutic drugs.

### Detection of Apoptosis and Proliferation in AAA

Apoptosis of SMCs in the aortic wall is one of the main cellular hallmarks of AA formation, making this process an interesting target for diagnostic imaging to visualize AA development. The SPECT/CT imaging probe ^99m^Tc-duramycin was developed to specifically target phosphatidylethanolamine (PE), which translocates from the inner to the outer cell membrane upon apoptosis (161). ^99m^Tc-duramycin enabled successful detection of SMC apoptosis at the site of aneurysm formation in AngII-infused mice. Considering the important role of SMC apoptosis in AA development, this imaging probe could be very useful for early detection of AA formation, but its predictive value should be further assessed.

In response to vascular injury, SMCs can undergo phenotypic switching to a synthetic, proliferative phenotype to promote remodeling of the vessel wall (162). Proliferation of SMCs is an early event in AAA and could therefore form a useful early marker for AAA detection. Proliferating cells can be targeted *in vivo* using the PET/CT radiotracer fluorine-18 fluorothymidine ([^18^F]FLT), which accumulates in proliferating cells (163). [^18^F]FLT signal increased during the active growth phase of AAA formation in AngII-infused *ApoE*^−/−^ mice and shows potential for early detection of AAA. Less probe uptake was observed in later phase AAA, where the growth phase plateaus, therefore this marker might be less useful for the prediction of aneurysm rupture.

### Detection of Microcalcification and Angiogenesis in TAA and AAA

Vascular calcification is a risk factor for developing cardiovascular disease and microcalcification of the aortic wall has been shown to be associated with AAA progression (164). Microcalcification is the deposition of calcium hydroxyapatite at the (sub)micrometer scale in the aortic wall and is associated with instability and rupture of plaques in atherosclerosis (165, 166). The bis-phosphonate NIRF probe OsteoSense-800 targets hydroxyapatite and enables *in vivo* visualization of microcalcification using FMT imaging. This probe was able to visualize microcalcifications in the ascending aorta of *Fbn1*^*C*1039*G*/+^ mice, a model for Marfan syndrome (167). Additionally, microcalcification can be visualized using fluorine-18-sodium fluoride (^18^F-NaF) PET/CT. ^18^F-NaF binds to calcium hydroxyapatite structures and correlates with AAA development and progression in AngII-infused *ApoE*^−/−^ mice (168). Exclusively abdominal aortas that showed early signals of microcalcification subsequently developed aneurysms, which shows the predictive potential of this marker for aneurysm development.

Angiogenesis, the formation of new blood vessels, is a process that is observed in both AAA and TAA tissue and is thought to contribute to inflammation as well as remodeling and weakening of the aortic wall (169, 170). CD105 is an important marker for angiogenesis and the ^64^Cu-labeled TRC105 Fab fragment specifically binds to CD105 (171). ^64^Cu-NOTA-TRC105-Fab enhanced PET imaging showed increased angiogenesis in a calcium phosphate-induced AAA mouse model and shows potential in the detection of AAA progression (171). Further research is necessary to confirm whether this probe can be used for prediction of aneurysm development and rupture.

Finally, several studies show the involvement of stem cells in AA pathogenesis, and stem cells were shown to be significantly elevated in AAA tissue from patients compared to control tissue. Interestingly, it has been shown in Ang-II infused *ApoE*^−/−^ mice that intravenous administration of mesenchymal stem cells can prevent aortic dilation (172). Stem cells possess several markers that could be exploited for specific targeting. Targeting stem cells for molecular imaging, therapy or even theranostics (combined diagnostics and therapeutics) would therefore be an interesting topic for future investigation.

## Clinical Trials in Molecular Aortic Aneurysm Imaging

Several imaging techniques that specifically target underlying molecular mechanisms of AA development are successfully being explored in mice. However, the clinical translation of molecular imaging for AA is relatively new and this process requires much time and is costly. Besides the necessity of human trials, testing for efficacy and accuracy outcomes, proper understanding of toxicology, bio-distribution, and circulation time analysis are of utmost importance to ensure patients safety. These difficulties secondarily clarify why only few of the markers that have been tested in mice, have presently been tested clinically.

Despite these challenges in clinical translation, some molecular markers have already been translated toward the clinical phase (Figure 4). However, due to the aforementioned challenges in clinical translation, it is crucial to select viable individuals/populations who are prospected to benefit the most. Consequently, current clinical studies on molecular imaging for AA ultimately aim to improve rupture risk assessment, trying to improve the main prevailing challenge in aortic repair today. And, as such, current research on these techniques are mainly being performed in context of AAA rather than TAA, due to the higher prevalence of AAA.

18-fluorodeoxyglucose (FDG) positron emission tomography (PET) based on an *in vivo* introduction of radioactively labeled glucose, of which cellular uptake can be detected with PET, is increasingly utilized in the last years (173). FDG-PET is used to detect sites of inflammation based on an increased glucose metabolic rate. A combination of wall inflammation and increased mechanical stress increases the risk of rupture, visualizing the grade of inflammation of the aortic wall, which could potentially translate to a higher pathogenicity and rupture risk. Various studies have explored the effectivity of FDG-PET for clinical AA risk-prediction, and found a correlation between wall degradation and the visualized inflammation (174, 175). Interestingly, this relationship was even more profound in patients with intraluminal thrombus (plaque deposition on aortic wall) (176), a finding which is associated with increased rupture risk (176, 177). Nevertheless, accurate AA risk prediction with FDG-PET remains uncertain, as few studies provide data demonstrating conflicting evidence regarding the associations of visualized inflammation with aneurysm dilatation (178, 179), and eventual rupture risk (180). Jalalzadeh et al. provided a systematic review of six studies of which 2 studies reported a significant correlation with FDG uptake and aortic inflammation, whereas 4 studies demonstrated no clear association (178).

Besides FDG, ^18^F-NaF (fluorine-18-sodium fluoride) that binds to calcium hydroxyapatite structures, has been studied in the clinic, demonstrating that high uptake is associated with aneurysmal disease and rupture/repair risk in humans too (181). Within *ex vivo* human aortic tissue, a couple of studies reported promising outcomes with regard to the radiotracer for α_v_β_3_ integrin and chemokine receptor 2 (CCR2) in *ex vivo* human aortic tissue, as autoradiography demonstrated specific binding of ^18^F-Fluciclatide and ^64^Cu-DOTA-ECL1i to α_v_β_3_ integrin and CCR2, respectively (182, 183). Furthermore, histopathological analysis demonstrated increased expression of CCR2 was co-localized with CD68+ macrophages. Although the prior study also found that, within rats, CCR2 tracer uptake in AAAs that subsequently ruptured (SUV = 1.31±0.14, p <0.005) demonstrated uptake nearly twice that of non-ruptured AAAs (SUV = 0.73±0.11). However, how effective these new tracer molecules will be in assessing a more accurate rupture risk prediction in human AAA remains an important factor for future investigation.

As proven in mouse models, ultrasmall paramagnetic particles of iron oxide, or ferumoxytol, was effective in detection of inflammation with PET/MR, and to help predict AA rupture (134). For a clinical study in 2011, aortic wall inflammation was imaged with ferumoxytol in a small group of AAA patients (184), and in 2017 a similar study was performed on a larger patient group, demonstrating a higher predictability of disease progression (185). In the latter study by Forsythe et al. in 2017, ferumoxytol enhancement was associated with reduced event-free survival for aneurysm rupture or repair (*P* = 0.0275), all-cause mortality (*P* = 0.0635), and aneurysm-related mortality (*P* = 0.0590) (185). However, after correcting for known risk factors such as maximum preoperative AAA diameter and smoking status, the addition of ferumoxytol enhancement to the multivariate model did not improve event prediction (c-statistic, 0.7935–0.7936).

Besides markers that have been tested in human AAA, other potentially viable markers that have been clinically tested in other diseases might hold potential in AAA too. Earlier it was shown that hypoxia positron emission tomography (PET) imaging agent ^64^Cu-diacetyl-bis (N4-methylthiosemicarbazone) (^64^Cu-ATSM) could be used for visualizing hypoxic cells in atherosclerosis in animal models (186), and recently this has been proven to be viable in human atheroma too (187). Although this study focused on carotid atherosclerotic plaques, the biological processes of atherosclerotic AA are similar, thus possibly holding potential in risk prediction too. As the aortic wall's primary source of oxygen is luminal blood flow, that gets disrupted in aortic tissue with intraluminal thrombus, it has been suggested that this is in part the source for aortic expansion and rupture (142). Secondarily highlighting the potential of this molecular marker.

Although MMPs have clearly shown to be convincing molecular markers for AAA degeneration in murine models (146), no clinical studies performing *in vivo* MMP imaging have yet been performed. Nevertheless, besides the fact that plasma levels of MMPs were demonstrated to be elevated in humans with AAA (188), Ducas et al. recently confirmed a correlation between *in vitro* human aortic tissue with intraluminal thrombus deposition and MMPs (142). Furthermore, in the last two decades, several fluorescent-labeled or radio-labeled MMP inhibitors have been developed as imaging agents for diseases, such as in myocardial infarction, intracranial aneurysms and multiple sclerosis (MS) (189), and should be clinically tested for AA in the future as well.

Besides the abovementioned biomarkers, molecular markers that have proven to be effective for rupture risk in the pre-clinical phase, targeting ECM proteins (type I/IV collagen and elastin), albumin, and immune cells, have yet to be tested clinically for practical use. Emphasizing that a great leap remains to be made toward the transition of human trials to test for clinical validity.

## Perspectives and Conclusions

Contemporary imaging techniques for AA risk prediction rely on visual parameters such as the maximum aortic diameter. Although radiological methods have improved over the years, risk prediction for AA ruptures through imaging remains suboptimal, resulting in a one-size-fits-all policy for intervention while a more tailored patient specific approach is desirable. Murine models for TAA and AAA continue to provide us with an in-depth understanding of the pathophysiological pathways at a cellular and molecular level that are consequential to aortic degeneration and AA formation. With the advancement of technology, newer imaging techniques are capable of targeting these molecular pathways involved in disease progression, potentially allowing the determination of disease progression not only in mice, but also in patients. Interestingly, these clinical applications might in return also lead to more detailed understanding of pathophysiological processes.

Within the pre-clinical setting, new potentially promising biomarkers such as collagen hybridizing peptide (190), targeting processes that are foundational in the pathophysiology of AA formation and rupture, will be subject to future study. However, forthcoming pre-clinical studies should be more attuned to the potential viability in clinical translation by focusing effectivity of biomarkers on rupture risk assessment. Notwithstanding, the current delay in clinical translation is in part due to the inability of creating an appropriate mouse model that replicates AA formation in humans. One of the biggest limitations is the difference in locations of AAs in murine models and humans, which may reflect regional differences in molecular composition, secondarily resulting in different mechanical properties. Furthermore, AAA murine models are induced chemically, and are consequentially unable to reproduce the complex nature of human AAA development. Improvement of the pre-clinical replica will lead to an enhanced anticipation whether successful pre-clinical markers will mirror its findings in humans as well. Additionally, to get a better view on the clinical applicability of molecular imaging markers, pre-clinical studies can be improved by assessing the predictability of markers in mouse models for AAA as well as TAA. Currently, most markers are being tested in mouse models for AAA, and especially considering the differences in underlying molecular mechanisms, it is useful to know whether imaging techniques can be applied for both AAA and TAA. Therefore, additionally, to get a better view on the clinical applicability of molecular imaging markers, pre-clinical studies can be improved by assessing the predictability of markers in mouse models for AAA as well as TAA.

Besides the assessment of the predictive value of biomarkers, novel molecular imaging techniques mentioned in this review can be particularly valuable for pre-clinical testing of the efficacy of potential interventional therapeutics. Molecular imaging of elastin in mice treated with IL−1β neutralizing antibodies allowed the monitoring of the response to the inflammatory treatment, which reduced aneurysm expansion, besides the prediction of AAA growth and rupture (133). This shows the potential of molecular imaging techniques to improve monitoring of the efficacy of interventional therapeutics, possibly also in a clinical setting. Additionally, a novel theranostic application using ultrasound imaging of VCAM-1-targeted microbubbles allowed simultaneous diagnostic imaging and targeted delivery of therapeutic drugs. This study demonstrates the potential of simultaneous targeting of molecules underlying AA development for molecular imaging as well as for therapy.

Future studies will have to evaluate whether novel radiological techniques utilizing molecular biomarkers could be of routine use in clinical practice and provide us with a more accurate and personalized risk prediction for AA disease. At present, the clinical literature does not yet report a feasible molecular marker that is independently associated with AA risk rupture. Accumulation or degradation of specific ECM proteins, increased MMP activity and immune cell infiltration have been shown to be highly correlated with AA development, progression, and even rupture risk in the pre-clinical phase, and, therefore potentially hold a predictive value for more accurate risk assessment for surgical intervention in the clinic as well. In order to achieve clinical applicability, prospective human trials need to be initiated. Although few biomarkers have already proven their association with disease progression in a few initial studies, it remains of importance to assess the clinical viability by providing a significantly more accurate risk prediction of AA rupture.

At first it will be essential to study which probes lead to highest sensitivity and specificity of rupture prediction within a given time frame. Pre-clinical studies already give insight into which markers could be most suitable. Specifically, probes targeting ECM proteins collagen and elastin as well as blood protein albumin were shown to successfully predict rupture risk in a mouse model of induced AAA. Furthermore, combined molecular imaging targeting elastin or collagen as well as immune cells showed superior results concerning AAA rupture prediction compared to molecular imaging of these targets individually. Markers such as MMPs and microcalcification hold predictive value concerning aneurysm growth, but whether they are also able to predict aneurysm rupture still has to be assessed. Thus, prioritizing translation of these markers is of utmost importance. Besides evaluation of independent molecular markers, future studies should assess the additional value of molecular imaging in which quantitative imaging analysis might play an important role. As AA development and progression is a multifactorial process, combined imaging of involved target molecules as well as visual parameters could provide us with a more comprehensive and complete view of the disease process and potentially allow for a more accurate risk assessment for AA development, progression, and even rupture risk. Leveraging computational methods and artificial intelligence that combine visual parameters with molecular imaging and the clinical presentation of the patient, shall potentially help lead toward even higher accuracy risk prediction of AA rupture. Furthermore, molecular markers that can be imaged with readily available imaging techniques in hospitals shall have a priority. Markers that need to be imaged with MRI, rather than by SPECT or CT, will be less practical due to the inherent time and cost of this imaging technique. As mentioned previously, ultrasound has been tested as theranostic molecular imaging modality within the pre-clinical setting, by utilizing microbubbles (160, 191). Furthermore, the use of ultrasound as a molecular imaging modality as clinical AA-risk assessment tool holds great potential, given its wide use and practicality. Currently, there have not been any clinical trials utilizing molecular imaging with ultrasound in context of AA. Thus, new promising molecular markers regarding clinical AA-risk assessment, shall require future investigation with ultrasound as the imaging modality.

Furthermore, other technologies surrounding molecular imaging should be assessed. As previously mentioned, recent studies have demonstrated that the use of a dual probe in AAA murine models that concurrently target markers both in the processes of ECM degradation alongside inflammatory activity, leads to higher sensitivity (80%) and specificity (90–100%) of risk prediction (130, 134). As aneurysmal disease (especially AAA) is a multifactorial environment of pathobiological activity within a degraded aortic wall, simultaneous insights in molecular pathways from different biological processes could provide us with a better understanding of the ongoing disease process and therefore allow us to provide more accurate risk prediction analyses. Combinations of probes with different pathophysiological processes should be assessed, to evaluate the most effective combinations for potential clinical use.

## Author Contributions

VR, SS, JB, IP, and JE contributed to conception and design of the study. VR, SS, and JH wrote the first draft of the manuscript. HV, JB, IP, and JE wrote sections of the manuscript. All authors contributed to manuscript revision, read, and approved the submitted version.

## Funding

This work was supported by NWO-FAPESP project 457002001 (IvdP, SS).

## Conflict of Interest

The authors declare that the research was conducted in the absence of any commercial or financial relationships that could be construed as a potential conflict of interest.

## Publisher's Note

All claims expressed in this article are solely those of the authors and do not necessarily represent those of their affiliated organizations, or those of the publisher, the editors and the reviewers. Any product that may be evaluated in this article, or claim that may be made by its manufacturer, is not guaranteed or endorsed by the publisher.
